# Dipeptidyl peptidase-4 inhibition with linagliptin prevents western diet-induced vascular abnormalities in female mice

**DOI:** 10.1186/s12933-016-0414-5

**Published:** 2016-07-08

**Authors:** Camila Manrique, Javad Habibi, Annayya R. Aroor, James R. Sowers, Guanghong Jia, Melvin R. Hayden, Mona Garro, Luis A. Martinez-Lemus, Francisco I. Ramirez-Perez, Thomas Klein, Gerald A. Meininger, Vincent G. DeMarco

**Affiliations:** Department of Internal Medicine, Division of Endocrinology, Diabetes and Metabolism, University of Missouri-Columbia School of Medicine, One Hospital Drive, Columbia, MO 65212 USA; MU Diabetes and Cardiovascular Research Center, University of Missouri, School of Medicine, Columbia, USA; Department of Medical Pharmacology and Physiology, University of Missouri, School of Medicine, Columbia, USA; Research Service, Harry S. Truman Memorial Veterans Hospital, Columbia, USA; The Dalton Cardiovascular Research Center, Columbia, USA; Boehringer Ingelheim Pharma, Ingelheim Am Rhein, Germany

**Keywords:** Pulse wave velocity, Atomic force microscopy

## Abstract

**Background:**

Vascular stiffening, a risk factor for cardiovascular disease, is accelerated, particularly in women with obesity and type 2 diabetes. Preclinical evidence suggests that dipeptidylpeptidase-4 (DPP-4) inhibitors may have cardiovascular benefits independent of glycemic lowering effects. Recent studies show that consumption of a western diet (WD) high in fat and simple sugars induces aortic stiffening in female C57BL/6J mice in advance of increasing blood pressure. The aims of this study were to determine whether administration of the DPP-4 inhibitor, linagliptin (LGT), prevents the development of aortic and endothelial stiffness induced by a WD in female mice.

**Methods:**

C56Bl6/J female mice were fed a WD for 4 months. Aortic stiffness and ex vivo endothelial stiffness were evaluated by Doppler pulse wave velocity (PWV) and atomic force microscopy (AFM), respectively. In addition, we examined aortic vasomotor responses and remodeling markers via immunohistochemistry. Results were analyzed via 2-way ANOVA, p < 0.05 was considered as statistically significant.

**Results:**

Compared to mice fed a control diet (CD), WD-fed mice exhibited a 24 % increase in aortic PWV, a five-fold increase in aortic endothelial stiffness, and impaired endothelium-dependent vasodilation. In aorta, these findings were accompanied by medial wall thickening, adventitial fibrosis, increased fibroblast growth factor 23 (FGF-23), decreased Klotho, enhanced oxidative stress, and endothelial cell ultrastructural changes, all of which were prevented with administration of LGT.

**Conclusions:**

The present findings support the notion that DPP-4 plays a role in development of WD-induced aortic stiffening, vascular oxidative stress, endothelial dysfunction, and vascular remodeling. Whether, DPP-4 inhibition could be a therapeutic tool used to prevent the development of aortic stiffening and the associated cardiovascular complications in obese and diabetic females remains to be elucidated.

**Electronic supplementary material:**

The online version of this article (doi:10.1186/s12933-016-0414-5) contains supplementary material, which is available to authorized users.

## Background

Increased arterial stiffness is a cardiovascular (CV) biomarker strongly associated with hypertension, diastolic heart failure, chronic kidney disease and stroke [1, 2]. Importantly, obese, insulin resistant and diabetic women develop vascular stiffness more frequently than men and this circumstance might account for the disproportionate incidence of cardiovascular disease (CVD) in insulin resistant women [3, 4]. The widespread consumption of diets high in refined carbohydrates and fat [western diet (WD)] is one of the driving forces behind the alarming growth in the incidence of obesity and insulin resistance [5, 6]. The progression to CVD in the setting of obesity and type 2 diabetes is likely initiated by endothelial dysfunction and several associated vascular abnormalities, including altered vascular tone, extracellular matrix remodeling and adventitial dysfunction, all of which could promote arterial stiffness [7, 8]. We recently tested this notion and reported that after 4 months of WD feeding, the aorta of normotensive female C57BL/6J mice exhibits altered nitric oxide (NO)-dependent vasodilation associated with aortic and endothelial stiffness, increased aortic wall thickening and fibrosis, oxidative stress and inflammation [9]. Recent reports from our laboratory indicate that therapies targeting vascular stiffness could potentially improve CV outcomes in insulin resistance models [7, 9]. In this regard, the use of dipeptidyl peptidase-4 (DPP-4) inhibitors may be of interest. DPP-4 is an exopeptidase that circulates in plasma and is also expressed on the surface of multiple cell types including endothelial and vascular smooth muscle cells, as well as immune cells [10]. Although DPP-4 inhibitors were developed to control hyperglycemia, it is now established that these inhibitors have effects beyond glycemic regulation [11]. The rationale for using DPP-4 inhibitors to target vascular stiffness relates to the potential of these compounds to improve endothelial function [11, 12]. Even though the beneficial effects of DPP-4 inhibition on the cardiovascular system have been explored in both animal models and in type 2 diabetic patients [11], to the best of our knowledge, the potential role of these compounds to target vascular stiffness in a female rodent model of insulin resistance due to over-nutrition without frank hyperglycemia or blood pressure elevation has not been studied. In the present investigation, we tested whether administering linagliptin (LGT), a long acting and specific DPP-4 inhibitor, to female C57BL/6 J mice fed a WD for 4 months, could ameliorate the development of WD-induced aortic and endothelial cell (EC) stiffness. Herein, we report that DPP-4 inhibition prevents the development of WD-induced aortic and EC stiffness in overweight female mice. The mechanism, in part, appears a consequence of preventing abnormalities in aortic endothelium-dependent vasorelaxation, oxidative stress, medial wall thickening and fibrosis, as well as expression of FGF-23 and Klotho.

## Methods

### Animal models

Three week old C57BL/6 J female mice were purchased from Jackson Laboratories (Bar Harbor, ME) and cared for in accordance with National Institutes of Health guidelines. All procedures were approved in advance by the Institutional Animal Care and Use Committee of the University of Missouri. Beginning at 4 weeks of age, mice were fed a WD consisting of high fat (46 %) and high carbohydrate as sucrose (17.5 %) and high fructose corn syrup (17.5 %) for 4 months (Test Diet 58Y1 with high refined carbohydrate and high fat, 5APC, Richmond, Indiana). Parallel groups of age-matched control mice were fed a modified control diet (CD) for the same period of time (Test Diet 58Y2). Mice were provided water ad libitum while housed in groups of four under a 12-hour/day illumination regimen. Based on a daily food intake of 0.1 g of chow per g body weight observed in a previous cohort of WD-fed mice, we added LGT (BI 1356; (R)-8-(3-aminopiperidin-1-yl)-7-but-2-ynyl-3-methyl-1-(4-methyl-quinazolin2-ylmethyl)-3,7-dihydro-purine-2,6-dione) [13] to mouse chow so that the final concentration in chow was 83 mg LGT kg^−1^ to achieve a dose and plasma level of approximately 8 mg kg^−1^ day^−1^ and approximately 50–100 nM, respectively [14]. Four groups of mice were utilized and they include mice fed a control diet without LGT (CDC), control diet with LGT (CDL), western diet without LGT (WDC) and western diet with LGT (WDL). Different sub-cohorts underwent the procedures described below.

### Body weight

To assess weight gained during the 4 month experiment, mice were weighed immediately prior to the start of the experiment at 4 weeks of age and before sacrifice.

### DPP-4 activity assay

DPP-4 activity assay was performed as previously described [15]. Briefly, blood was collected at the time of killing in EDTA tubes and plasma was stored at −80 °C. 20 μL plasma was diluted in DPP-4 assay buffer and substrate, 200 M H-Ala-Pro-AFC (I-1680; Bachem), was added. Fluorescence was measured with a Synergy Microplate Reader at excitation wavelength of 405 nm and emission wavelength of 535 nm [15].

### Aortic stiffness by in vivo pulse wave velocity (PWV)

Doppler ultrasound (Indus Mouse Doppler System, Webster, TX) was performed under isoflurane anesthesia, according to a previously established protocol [9], to evaluate PWV for in vivo determination of arterial stiffness. PWV is affected by age, blood pressure, and heart rate. Consequently, PWV is reported both in m/s (unadjusted), as well as normalized to heart rate (HR). Limiting the WD feeding period to 4 months allowed us to avoid any effects of increased blood pressure on vascular stiffening as 4 months of WD does not induce an elevation of blood pressure in female C57BL/6J mice [9].

### Ex vivo vasomotor responses in the aorta

Aortic vasomotor responses were examined as previously described [9]. Briefly, vessels were preconstricted with U46619 (100 nM) and responses to acetylcholine (ACh) (1 nm to 100 μmM), and to insulin (INS: Novolin R, Novo Nordisk; 0.1–300 ng/mL) were assessed by cumulative addition of agonist to an isolated vessel bath as previously described [9]. At the end of each experiment, the PSS bath solution was replaced with Ca^2+^-free PSS to determine maximal relaxation.

### Atomic force microscopy (AFM) for force measurement and stiffness calculation

AFM was used to evaluate stiffness of ECs in enface aortic preparations of the thoracic aorta as previously described [9]. Stiffness (elastic modulus) of the EC surface was measured by AFM using a nano-indentation protocol [9].

### Aortic remodeling and protein quantification

A segment of thoracic aorta was fixed in 3 % paraformaldehyde, dehydrated, paraffin embedded, and transversely sectioned in 5 µm slices. To evaluate peri-aortic fibrosis, as previously described, the sections were stained with picro sirius red. To evaluate the medial thickness the sections were stained with Verhoeff-von Gieson [9]. The pink color intensities and areas were quantified as “average gray scale intensities” in five images per mouse with MetaVue software. Aortic oxidative stress was assessed by quantifying brown color in the endothelium, media and adventitia of the aorta which is indicative of 3-nitrotyrosine formation (1:150 dilution; Millipore, Billerica, MA), as previously described [16]. The expression level of fibroblast growth factor-23 (FGF-23) (1:50 dilution; Novus NBP1-55830), klotho (1:100 dilution; Novus NBP1-76511) and advanced glycation end-products (AGE) (1:100 dilution; Abcam ab23722) was determined by quantifying of signal intensities on the fluorescent images on the different components of aorta as “average gray scale intensities”.

### Transmission electron microscopy (TEM)

TEM was performed as previously described using a JEOL 1400-EX TEM to determine ultrastructural changes associated with increased vascular stiffness [17]. Briefly, aortic samples were collected and fixed in 2 % glutaraldehyde/2 % paraformaldehyde followed by secondary fixation with 1 % osmium tetroxide, embedded in Epon-Spurr’s resin, sectioned at 85 nm, and stained with uranyl acetate/Sato’s triple lead stain.

### Statistical analysis

Results are reported as the mean ± SE. Statistical analysis was primarily by two-way ANOVA followed by post hoc t tests (Bonferroni) to examine differences in outcomes between mice fed CD or WD and administered LGT in mouse chow or with no LGT in the chow (Sigma Plot 13.0, Systat Software). Aortic dilator responses are presented as percent maximal relaxation, calculated as [(Fb−Fd)/(Fb−Fmin)]*100, where Fd is force after a drug intervention, Fb is baseline force, and Fmin is minimal force obtained during passive conditions.

## Results

### Body weight, plasma DPP-4 activity and AGE

**Fig. 1:**
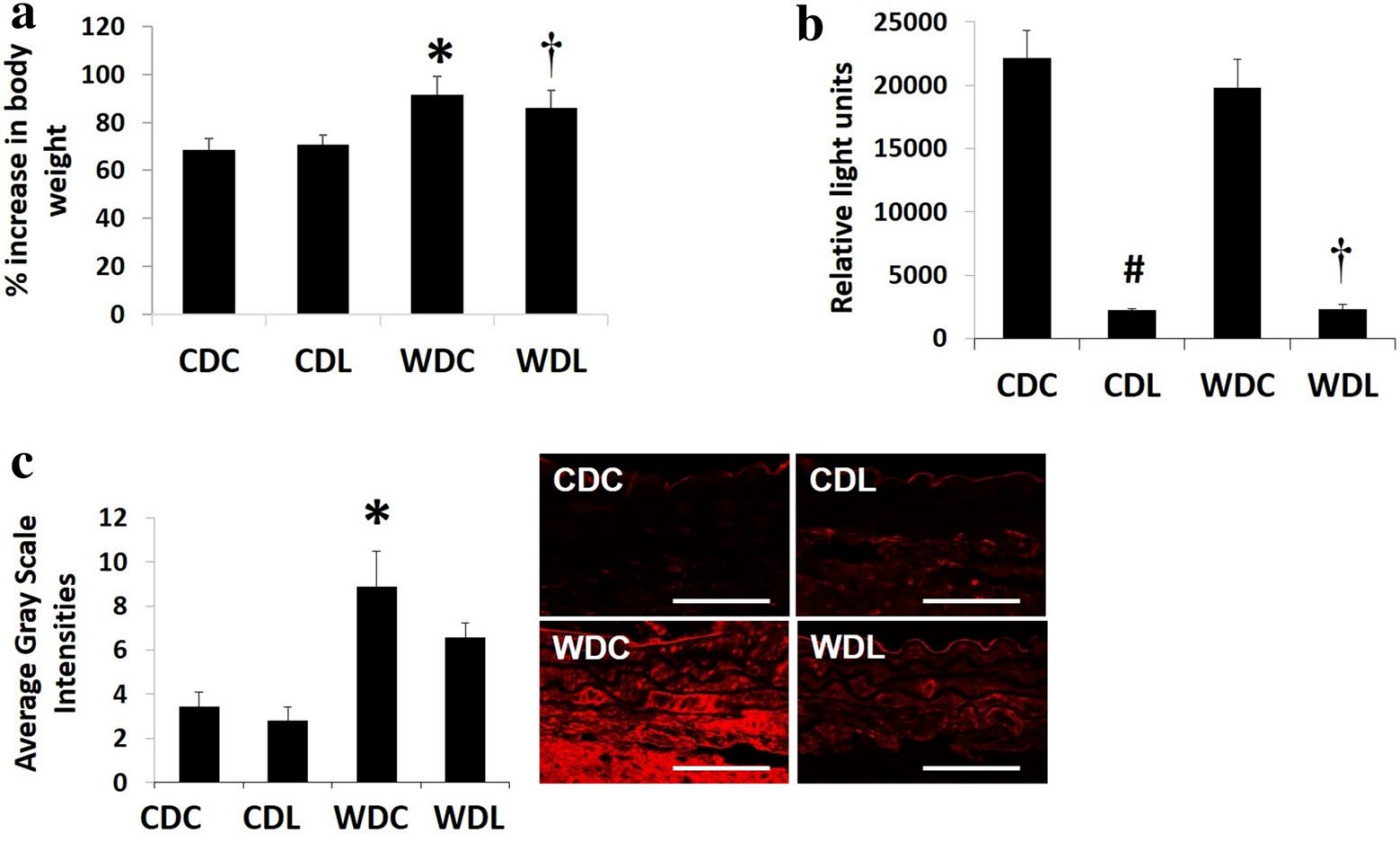
Linagliptin (LGT) has neutral effects on body weight and aortic advanced glycation end-products (AGE). **a** WD feeding for 4 months resulted in significant weight gain in both the cohorts. **b** Plasma DPP-4 activity was significantly decreased with LGT treatment. **c** AGE immunostaining in aorta was significantly increased in WDC. LGT treatment did not decrease it significantly. Quantification and representative images shown. Values are mean ± SE. *CDC* control diet control, *CDL* control diet linagliptin, *WDC* western diet control, *WDL* western diet linagliptin. Post-hoc comparisons within a time point; *p < 0.05 CDC vs WDC; ^#^p < 0.05 CDC vs CDL; ^†^p < 0.05 WDC vs WDL. *Scale bars* represent 50 mμ

Body weights of 20-week old WDC and WDL mice were of similar weights and heavier compared to those of their respective lean counterparts (Additional file 1: Table S1; Fig. 1a). Percent body weight gain during the 4 month study period was 71 ± 4 and 93 ± 6 % for CDC and WDC (p > 0.05), respectively, and 74 ± 7 % and 88 ± 7 % for CDL and WDL (p > 0.05), respectively. Thus, DPP-4 inhibition had no significant effect on body weight (Additional file 1: Table S1; Fig. 1a) within each dietary regime during the test period. As expected, DPP-4 plasma activity was significantly decreased in both groups treated with LGT without a diet effect (Fig. 1b). As AGE have been associated with vascular stiffness in some models of insulin resistance [18, 19], we examined the presence of AGE in the aortic wall (Fig. 1c). WD significantly increased the accumulation of AGE in the vascular wall, and this was not affected by administration of LGT.

### DPP-4 inhibition prevented WD-induced aortic and EC stiffness

#### In vivo

**Fig. 2:**
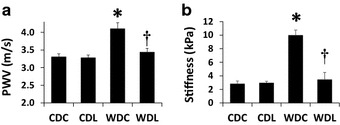
LGT prevents development of in vivo aortic stiffening, as well as endothelial stiffening in ex vivo aortic explants. **a** Pulse wave velocity (PWV) measured after 4 months on experimental diets. **b** Force measurements were acquired by interaction between a cantilever tip and the EC surface of aortic explants from mice after 4 months on WD. Values are mean ± SE. *CDC* control diet control, *CDL* control diet linagliptin, *WDC* western diet control and *WDL* western diet linagliptin. Post-hoc comparisons within a time point; *p < 0.05 CDC vs WDC; ^†^p < 0.05 WDC vs WDL

PWV was determined in mice following four months on CD or WD (Fig. 2a). PWV did not vary significantly in CDC mice (Fig. 2a; Table 1). PWV was elevated in the WDC group compared to CDC (p < 0.001) and administration of LGT prevented the WD-induced increase in PWV (p < 0.001) (Fig. 2a; Table 1). No differences in PWV were observed between CDL and WDL groups (p = 0.363). Normalization of PWV to HR yielded similar results as shown in the adjusted PWV analysis (Table 1).

**Table 1:** Aortic stiffness in untreated western diet (WD)-fed mice compared to untreated control diet (CD) fed mice as indicated by determinations of aortic pulse wave velocity (PWV)

Pulse wave velocity	Main effect	p value	CDC [6]	CDL [8]	WDC [10]	WDL [10]
4 months PWV (m s^−1^)	Diet	0.001	3.31 ± 0.08	3.28 ± 0.07	4.11*^†^ ± 0.16	3.44 ± 0.11
	Treatment	0.001				
	Interaction	0.017				
Heart rate (bpm)	Diet	0.036	442 ± 22	439 ± 26	397* ± 12	415 ± 5
	Treatment	0.639				
	Interaction	0.496				
4 months PWV (m s^−1^ HR^−1^ 100)	Diet	0.006	0.76 ± 0.04	0.77 ± 0.05	1.05*^†^ ± 0.06	0.83 ± 0.03
	Treatment	0.091				
	Interaction	0.072				

Also shown, is heart rate (HR) and PWV normalized to HR. Administration of linagliptin (WDL) prevented the increase in PWV observed WD-fed mice (WDC). Sample sizes are noted in parentheses. Values are mean ± SE

*CDC* control diet control, *CDL* control diet linagliptin, *WDC* western diet control and *WDL* western diet linagliptin. Post-hoc comparisons

* p < 0.05 CDC vs WDC; ^†^ p < 0.05 WDC vs WDL

#### Ex vivo

We recently reported increased EC stiffness in aortic explants utilizing AFM after 4 months of WD in female mice [9]. Similarly, in this investigation we observed increased surface stiffness in the EC from WD-fed mice (p < 0.05 CDC vs. WDC) and this finding was prevented by LGT administration (p < 0.05 for WDC vs. WDL and p > 0.05 for CDC vs. WDL) (Fig. 2b).

### DPP-4 inhibition improves endothelial dependent responses in the aorta

**Fig. 3:**
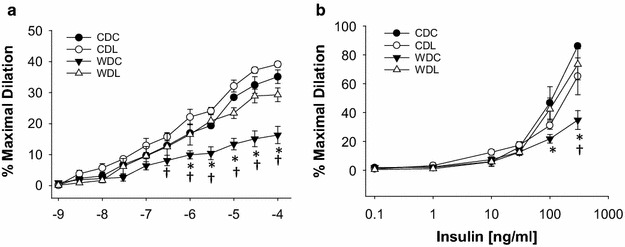
Vasodilator responses of isolated aortic rings to the endothelium-dependent dilators acetylcholine (**a**) and insulin (**b**). Values are mean ± SE. *CDC* control diet control, *CDL* control diet linagliptin, *WDC* western diet control and *WDL* western diet linagliptin. Post-hoc comparisons within a time point; *p < 0.05 CDC vs WDC; ^†^p < 0.05 WDC vs WDL

We recently reported that 4 months of WD feeding in female C57BL/6J mice induced impaired protein kinase B (Akt)/endothelial nitric oxide synthase (eNOS) signaling and this was associated with impaired aortic endothelium-dependent vasodilation [9]. Herein, we evaluate Akt/eNOS signaling in aortic rings by functional assay to examine the vasodilatory responses to ACh and insulin. Four months of WD feeding resulted in impaired aortic endothelium-dependent vasodilatory responses to ACh and insulin in the WDC compared to CDC (Fig. 3a, b). These defects were prevented in the WDL group (Fig. 3a, b).

### DPP-4 inhibition prevents vascular remodeling

**Fig. 4:**
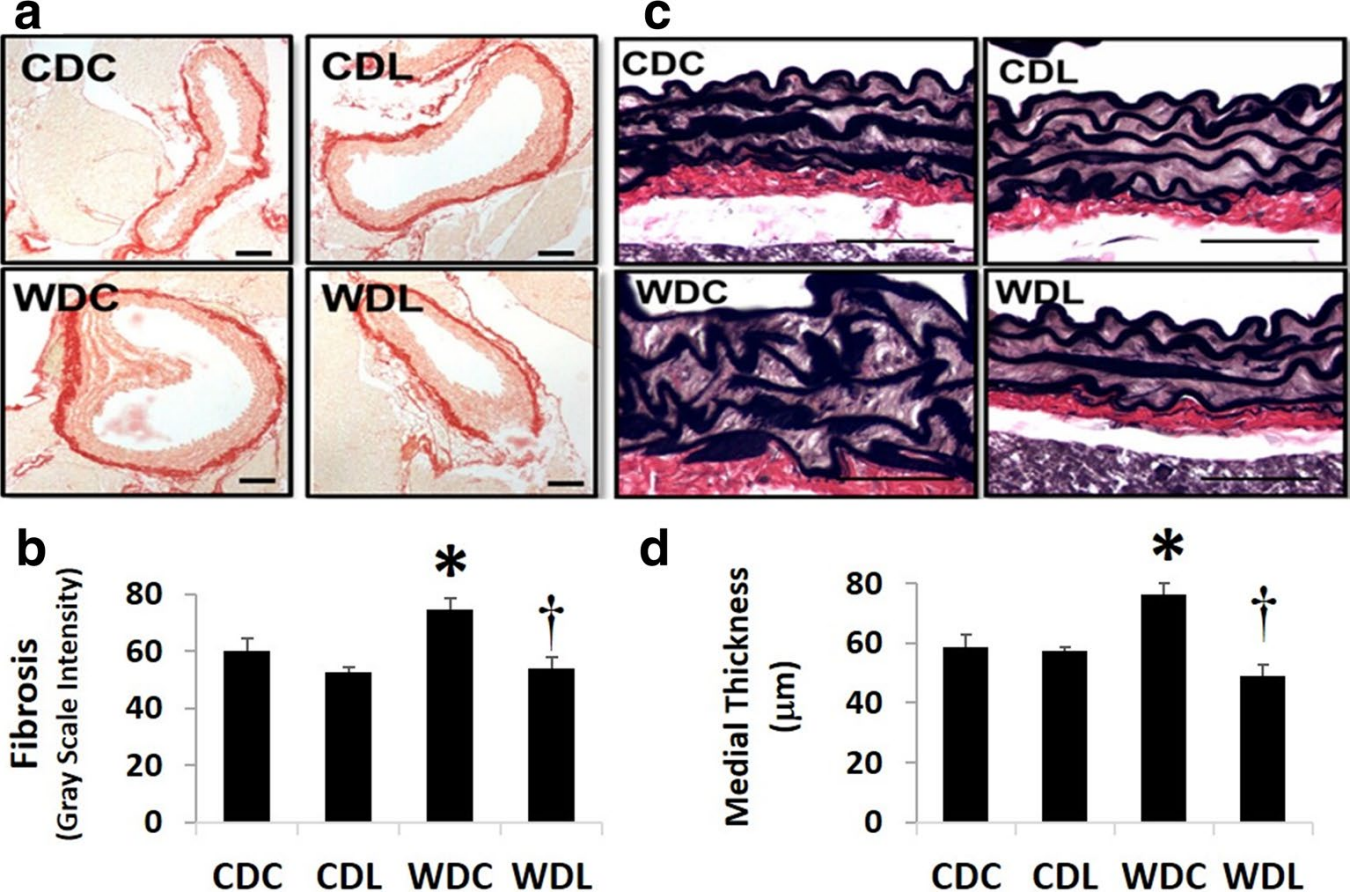
WD feeding causes **a**, **b** peri-aortic fibrosis and (**c**, **d**) medial thickening which is ameliorated by the DPP-4 inhibitor, LGT. **a** Picro sirius red and **b** Verhoeff-von Gieson staining. Values are mean ± SE. *CDC* control diet control, *CDL* control diet linagliptin, *WDC* western diet control and *WDL* western diet linagliptin. Post-hoc comparisons within a time point; *p < 0.05 CDC vs WDC; ^†^p < 0.05 WDC vs WDL

We have previously shown that WD feeding results in increased aortic remodeling. Similarly, in the present investigation, aortic medial thickness and fibrosis were significantly increased with WD feeding compared to CDC (Fig. 4a–d). Importantly, aortic fibrosis and increased medial thickness were prevented with DPP-4 inhibition (Fig. 4a–d).

### DPP-4 inhibition prevents the increase in WD-induced ultrastructure changes in EC

**Fig. 5:**
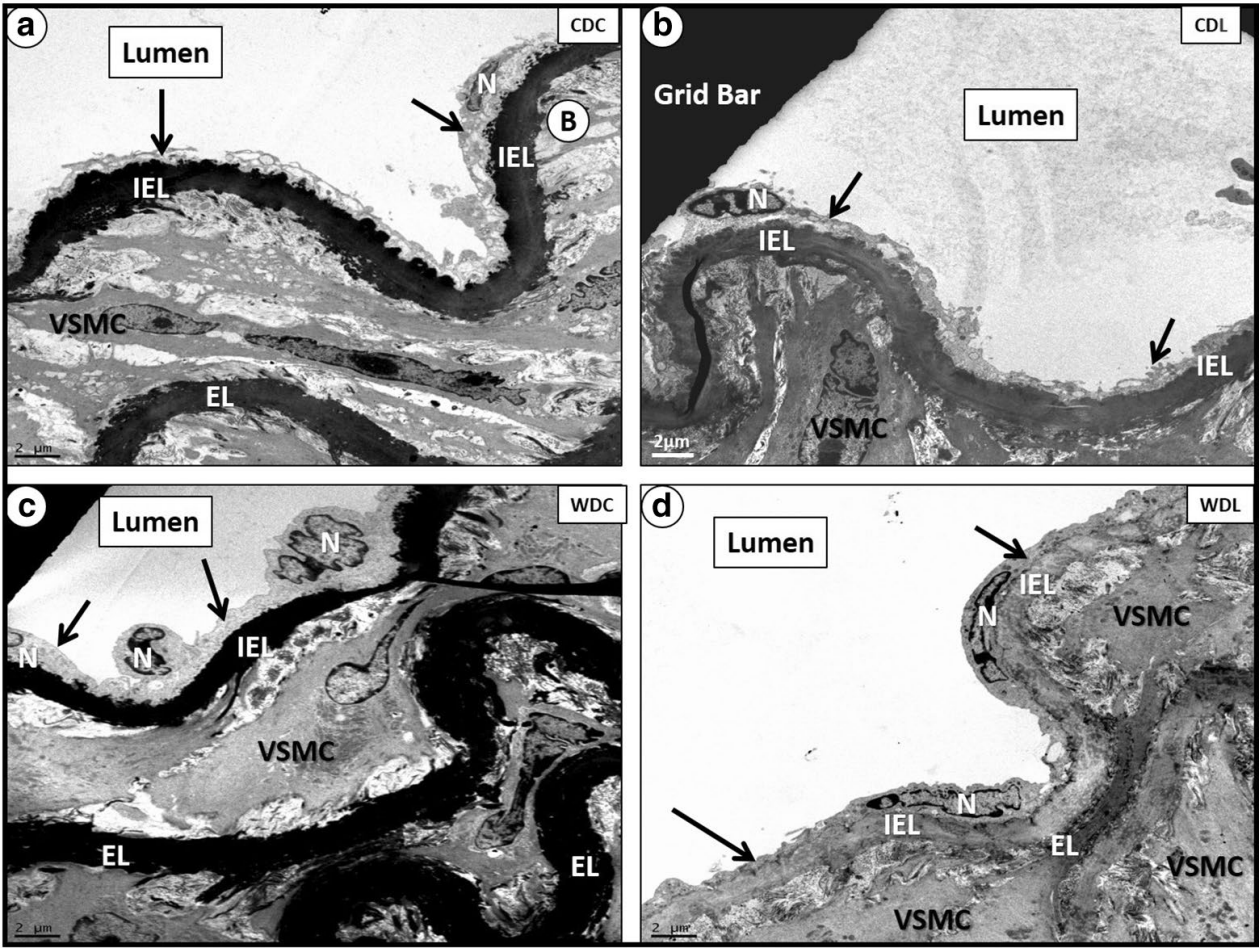
LGT treatment ameliorates loss of luminal EC cytoplasmic elongation and nuclear contraction induced by WD. **a**, **b** depict CDC and CDL cohorts with normal EC characteristics; **c** depicts contracted cytoplasm and nuclei of EC in WDC; **d** demonstrates the absence of luminal EC swelling, edema and contracted cytoplasm and nuclei seen in **c**. Magnification ×800; *bar* 2 µm. *EL* elastic lamina, *IEL* internal elastic lamina, *N* nucleus of luminal endothelial cell, *VSMC* vascular smooth muscle cell

Increased activation of the contractile apparatus of EC has been associated with the genesis of vascular stiffness [20]. In this study, we examined the ultrastructural characteristics of the aorta in the different experimental groups (Fig. 5a–d). WD was associated with loss of luminal endothelial cytoplasmic elongation and nuclear contraction and these abnormalities were prevented in the WDL group (Fig. 5c, d).

### DPP-4 inhibition prevents oxidative stress

**Fig. 6:**
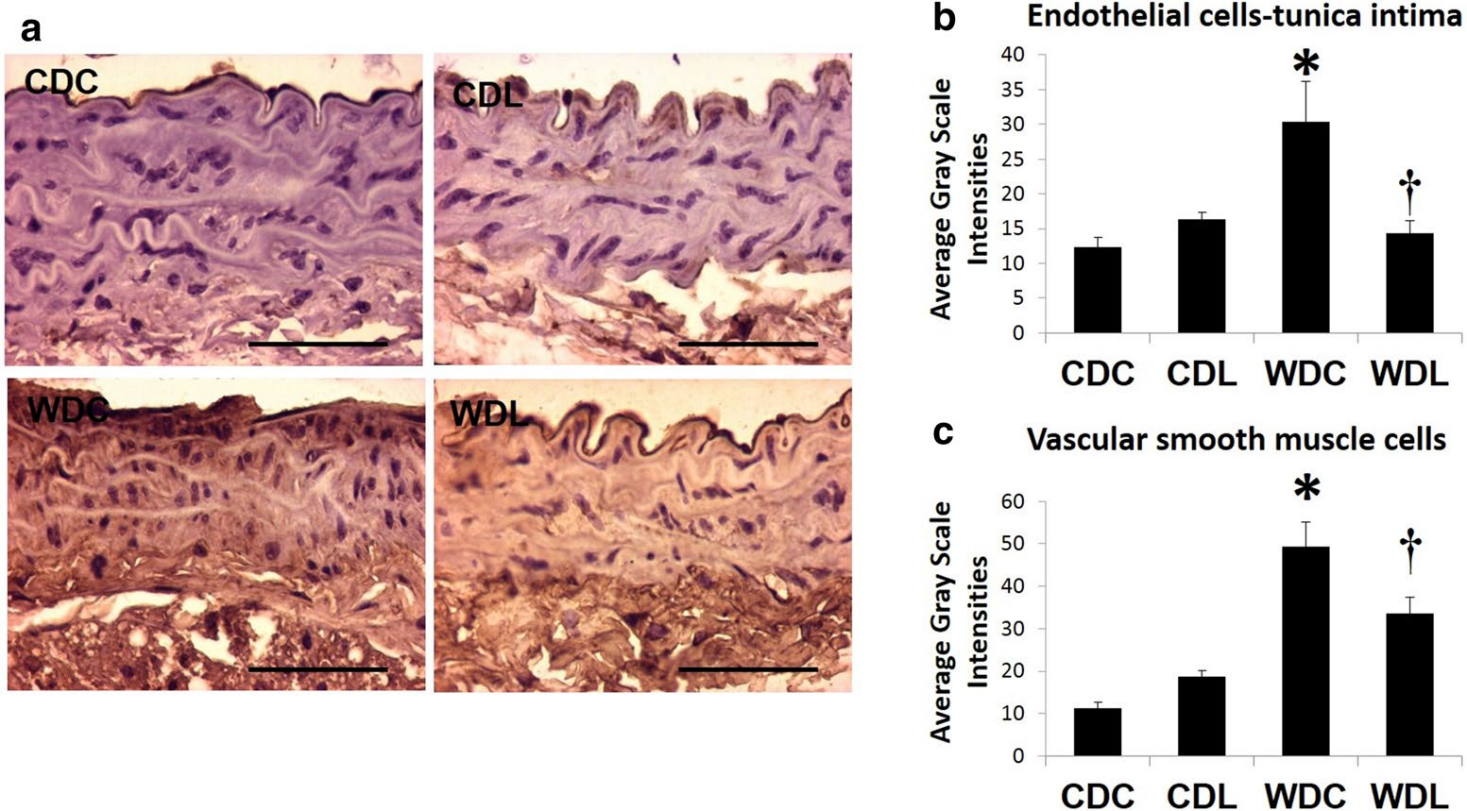
WD feeding induced aortic oxidative stress is ameliorated with DPP-4 inhibition. **a** 3-nitrotyrosine staining; **b** EC 3-nitrotyrosine; **c** VSMC 3-nitrotyrosine. Values are mean ± SE. *CDC* control diet control, *CDL* control diet linagliptin, *WDC* western diet control and *WDL* western diet linagliptin. Post-hoc comparisons within a time point; *p < 0 0.05 CDC vs WDC; ^†^p < 0.05 WDC vs WDL

We have previously shown that vascular stiffness induced by WD feeding in females was associated with increased vascular oxidative stress [9]. Here in we showed that WD increased aortic oxidative stress assessed by the presence of 3-nitrotyrosine immunostaining (Fig. 6). Importantly, DPP-4 Inhibition ameliorated aortic oxidative stress by normalization of 3-nitrotyrosine induced by WD feeding (Fig. 6).

### DPP-4 inhibition prevents WD-induced changes in FGF-23 and Klotho expression in the vascular wall

**Fig. 7:**
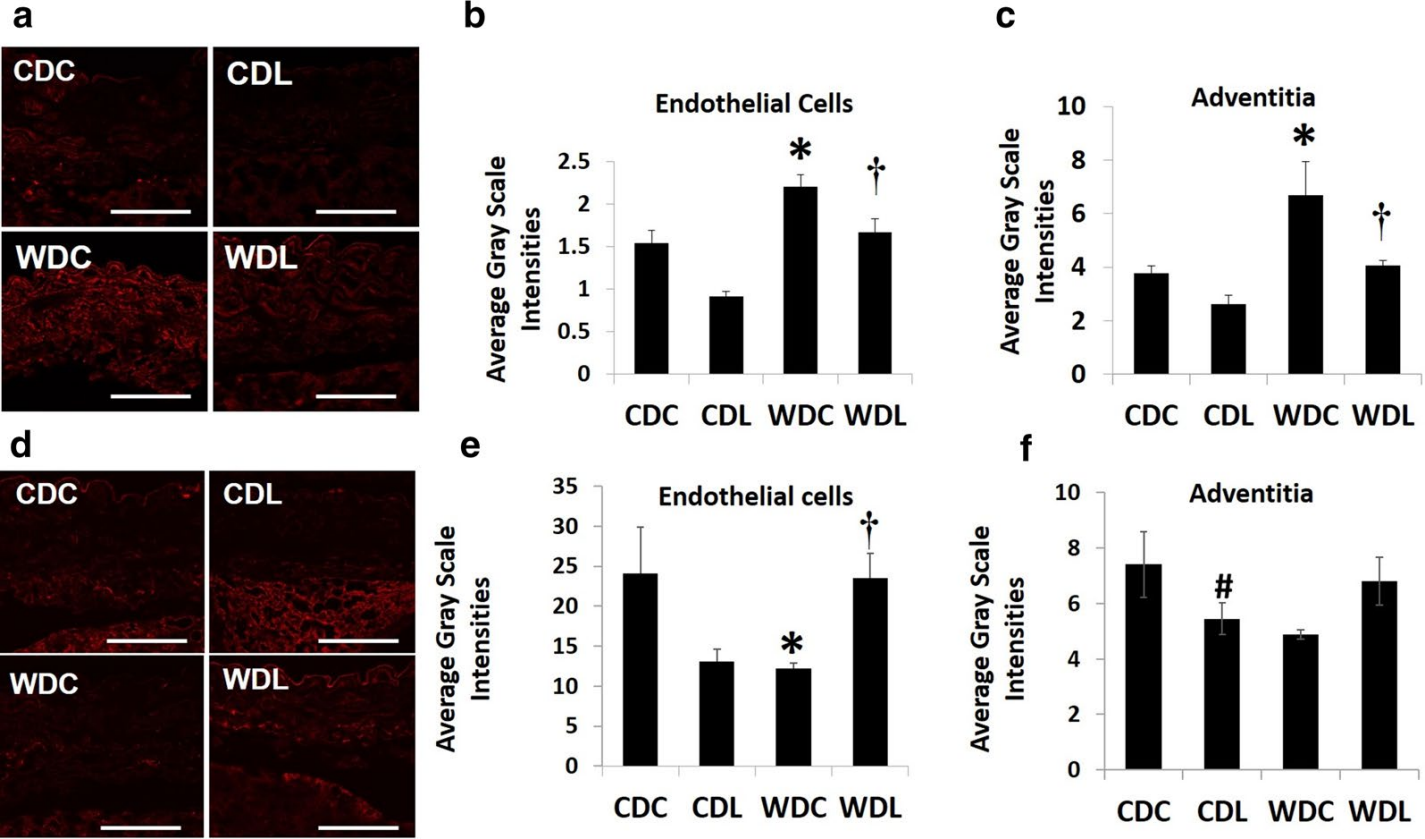
WD feeding induced changes in FGF-23 and Klotho expression are restored by DPP-4 inhibition. **a** FGF-23 staining; **b** Endothelial FGF-23; **c** Adventitia FGF-23; **d** Endothelial klotho staining; **e** Endothelial klotho staining **f** Adventitia klotho staining; Average gray intensities in the different cohorts. Values are mean ± SE. *CDC* control diet control, *CDL* control diet linagliptin, *WDC* western diet control and *WDL* western diet linagliptin. Post-hoc comparisons within a time point; *p < 0 0.05 CDC vs WDC; ^†^p < 0.05 WDC vs WDL; ^#^p < 0.05 CDC vs CDL. *Scale bars* represent 50 mμ

FGF-23 is associated with arterial stiffness and increased risk of CVD [21–23]. We have recently shown that this WD feeding paradigm results in increased FGF23 in the vascular wall [24]. Furthermore, decreased Klotho expression, another protein involved in vascular calcification, is known to exacerbate diet-induced vascular stiffness [9]. Herein we examined whether WD induces changes in FGF-23 and Klotho expression in the aorta, and if so, whether DPP-4 inhibition prevented that change (Fig. 7). As anticipated, we observed a significant increase in FGF-23 in the aortic endothelium and adventitia of the WDC group compared with CDC, and this increase was prevented in the WDL group (Fig. 7a–c). In addition, Klotho expression was significantly decreased by WD feeding and DPP-4 inhibition prevented this deficiency in the vascular wall (Fig. 7d–g).

## Discussion

Collectively, the results of this investigation support the hypothesis that DPP-4 activation plays an important role in the development of aortic stiffness in female mice in the setting of WD-induced insulin resistance. Furthermore, we present evidence that DPP-4 inhibition prevented female C57BL/6 J mice from developing WD-induced aorta stiffening, remodeling and dysfunction. We previously reported that WD feeding for 4 months does not increase blood pressure in female C57BL/6J [9]. This previous finding enabled us to design an experiment that factored out a potential blood pressure effect to explain the CV protective effects of DPP-4 inhibition.

Beneficial vascular effects of DPP-4 inhibition have been attributed to both direct effects of DPP-4 inhibition and to the collateral increase in glucagon like peptide-1 (GLP-1) availability [10]. In the vessel, DPP-4 is located in the cytoplasmic membrane of both EC and vascular smooth muscle cells (VSMC). In EC, DPP-4 expression is increased in models of diabetes [25] and in VSMC, DPP-4 expression is preferentially increased in conditions of vascular remodeling [11]. Importantly, the dose of LGT used in our investigation significantly inhibits DPP-4 activity [12, 14, 26].

Our group previously showed that 4 months of WD feeding in female mice resulted in increases in aortic stiffness, measured non-invasively, as well as EC stiffness, measured ex vivo [9]. Results of this study using a separate cohort of mice were nearly identical with our previous report and further demonstrate aortic and EC stiffening after 4 months of WD feeding. Notably, DPP-4 inhibition prevented the development of aortic stiffness, both in vivo and ex vivo, observed in untreated WD-fed mice. Although previous reports demonstrate that DPP-4 inhibitors decrease cardiac stiffness [12, 27, 28], to the best of our knowledge this is the first report demonstrating protection from vascular stiffening in a clinically relevant non-diabetic female model of over-nutrition.

Vascular stiffness is determined by EC and VSMC properties, as well as by extracellular matrix and adventitial characteristics [7]. In our experiments, DPP-4 inhibition in the WD-fed cohort prevented the development of impaired endothelial-dependent vasodilation, an effect in the aorta that is known to be largely NO-dependent [29, 30]. In this regard, DPP-4 inhibitors exert direct effects on vascular tone independently of GLP-1 [11]. The DPP-4 inhibitors, LGT and alogliptin, in particular, exert vasodilatory effects in the aorta in the setting of enhanced inflammation and oxidative stress and these effects were mediated by the NO/cGMP pathway [14]. We previously reported a similar improvement in endothelium-dependent vasodilation in skeletal muscle arterioles with LGT administration in a model of obesity and insulin resistance [12]. More recently we showed that impaired NO signaling accompanies increased vascular oxidative stress in our WD-fed mouse model [9]. Thus, it is likely that a decrease in oxidative stress in our WD-fed female mice administered a DPP-4 inhibitor, helped to preserve normal aortic vaso-relaxation by preventing NO scavenging by free radicals, such as superoxide, as evidenced by a decrease in 3-nitrotyrosine staining in the aorta compared to untreated mice [7, 31]. In a different model of obesity and insulin resistance, LGT also ameliorated oxidative stress associated with cerebral ischemia independently of glycemic control [32], findings that further support the concept that the antioxidant properties of LGT may be vasculoprotective.

Likewise, the beneficial role of DPP-4 inhibition in restoring endothelial-dependent vasodilation in conditions of increased oxidative stress has been documented in other murine models [33, 34]. It should be noted that even though the magnitude of the aortic vasodilatory responses reported in this investigation seem low, similar results in mice have been reported from other laboratories [35–37]. Furthermore, our ultra-structural findings suggest that DPP-4 inhibition reverses the loss of luminal endothelial cytoplasmic elongations and nuclear contraction associated with WD feeding. Additionally, our findings indicate that the beneficial effects of the DPP-4 inhibition are not limited to the endothelium. Here, we observed that DPP-4 inhibition decreases medial thickening and fibrosis related to WD-feeding. The vascular anti-fibrotic role of DPP-4 inhibitors has been reported previously in models of atherosclerosis and neo-intima injury [34, 38].

FGF-23 is elevated in conditions of insulin resistance and obesity [39, 40]. Importantly, FGF-23 levels are associated with impaired vascular relaxation, vascular calcification and stiffness [22, 40, 41]. Moreover, FGF-23 is expressed in the vasculature [42] and has been shown to enhance oxidative stress and induce inhibition of NO-dependent vasodilation [43]. We recently reported that female WD-fed mice exhibit increased FGF-23 levels and this was associated with impaired aortic endothelial function, increased aortic PWV and increased oxidative stress [24]. FGF-23 effects may be modulated, in part, via Klotho [44]. In this regard, Klotho deficiency was recently reported to accelerate aortic stiffening in high fat fed mice [45]. Whether WD leads to a deficiency in Klotho expression has not been examined previously. Herein, we explored a possible contribution of FGF-23 and Klotho to the genesis of vascular stiffening, endothelial dysfunction and oxidative stress by testing whether DPP-4 inhibition prevents the increase in aortic FGF-23 expression and modulates Klotho expression in the aorta of WD-fed mouse model. In the present work, FGF-23 expression was increased in the aorta of WD-fed mice and this was prevented with DPP-4 inhibition. Furthermore, the expression of Klotho was decreased in the aorta of WD-fed mice relative to control mice and this deficiency was prevented with administration of LGT. In this regard and relevant to our findings, others have reported that Klotho deficiency results in increased production of reactive oxygen [46], while its over-expression ameliorates oxidative stress [47]. Since oxidative stress has been implicated in the impairment of vascular relaxation and bioavailable NO, the prevention of abnormal expression of FGF23 and Klotho by DPP-4 inhibition in the vasculature may represent a new mechanism to explain the efficacy of DPP-4 inhibition in preventing the appearance of vascular stiffness. Whether FGF-23/Klotho signaling in the vasculature, in concert with its vascular receptors and cofactors, directly modulates vascular function or influences vascular function via regulation of putative effects on mineral metabolism is unclear.

In the present investigation we did not observe that LGT prevented or reduced WD-induced weight gain suggesting that the efficacy of LGT in preventing WD-induced aortic stiffening, impaired vasoreactivity and abnormal remodeling did not result from beneficial weight loss. Some rodent studies have documented a similar neutral effect of LGT administration on body weight in association with improvement in cardiovascular function and structure [25, 48]. On the other hand, higher doses of LGT led to modest weight loss in rats fed a high fat diet [49]. Importantly clinical trials have supported the notion of weight stability with LGT treatment [50–52]. One of the limitations of the present investigation is that we did not directly evaluate glucose homeostasis or insulin resistance. Nevertheless, we have previously demonstrated that this diet paradigm in female C57BL/6J mice does not result in frank hyperglycemia while it manifests significant whole body insulin resistance evaluated by the hyperinsulinemic euglycemic clamp [53]. Herein, we further explored the possibility that DPP-4 inhibition reduces the anticipated increase in expression of AGE products as AGE/RAGE signaling can result in a pro-inflammatory and pro-oxidant environment [54]. Furthermore, AGE can increase expression of DPP-4 in EC [55]. In the present investigation, we documented increased AGE presence in the vascular wall of WD-fed females, and this was not prevented with DPP-4 inhibition. This suggests that the efficacy of DPP-4 inhibition in the vasculature of WD-fed female mice occurs without significant changes in AGE. To the best of our knowledge this is the only study demonstrating that a DPP-4 inhibitor prevents development of diet-induced vascular stiffness. Further studies are needed to clarify if other DPP-4 inhibitors share these beneficial effects.

Despite abundant preclinical evidence of cardiovascular benefits of DPP-4 inhibitors, including results presented herein, meta-analyses of clinical data raise concerns regarding evidence of increased risk of heart failure with administration of certain DPP-4 inhibitors [56]. On the other hand, other recent analyses have produced differing conclusions regarding heart failure risk assessment [57]. In the SAVOR-TIMI 53 trial the use of saxagliptin was associated with increased risk of heart failure [58]. Importantly, the risk was higher in the subjects with previous heart failure, elevated natriuretic peptides and impaired renal function [59]. In the EXAMINE trial that explored the use of alogliptin in type 2 diabetics with post-acute coronary events there was no increased risk of major CV events [60]. Nevertheless, a recent post hoc analysis of the EXAMINE trial did find a statistically significant increase in hospitalization rate from heart failure in the group of subjects without heart failure history [61]. On the contrary, the sitagliptin TECOS trial did not find differences in the rate of hospitalization for heart failure between the treatment and the control group [62]. Similarly, a recent pre-specified patient-level pooled analysis of available trials of LGT did not report an association between the DPP-4 inhibitor and increased CV risk (including heart failure) [63]. Results from two ongoing trials using LGT, the CAROLINA (NCT01243424) and CARMELINA (NCT01897532), will further clarify the role and safety of this agent in regard to diabetic CVD.

To summarize, the current investigation demonstrates that DPP-4 inhibition prevents abnormal increases in vascular stiffness, aortic fibrosis, oxidative stress and FGF-23/Klotho expression induced by a WD in female mice and these benefits occur independent of AGE reduction. Our findings have clinical relevance as obese diabetic women are more frequently affected by increased vascular stiffness that likely promotes higher incident CVD compared to their male counterparts. Ultimately, only the results of well-designed clinical trials will clarify the role of LGT in the management of diabetic CVD.

## Additional file

10.1186/s12933-016-0414-5 Body weights of C57BL/6J mice fed a western diet (WD) high in fat and high fructose corn syrup compared to mice fed a control diet (CD). WD induced significant increases in body weight in WDC and WDL compared to their respective control groups. Administration of linagliptin (L) had no effect on body weight.

## Abbreviations

DPP-4: dipeptidylpeptidase-4
WD: western diet
LGT: linagliptin
PWV: pulse wave velocity
AFM: atomic force microscopy
CD: control diet
CV: cardiovascular
CDC: control diet without LGT
CDL: control diet with LGT
WDC: western diet without LGT
WDL: western diet with LGT
Akt: protein kinase B
eNOS: endothelial nitric oxide synthase
Ach: acetylcholine
