# Supplementary material for: Dipeptidyl peptidase-4 inhibition with linagliptin prevents western diet-induced vascular abnormalities in female mice
Source: Cardiovasc Diabetol. 2016 Jul 8;15:94. doi: 10.1186/s12933-016-0414-5 (PMC4938903; doi:10.1186/s12933-016-0414-5)
Supplement: Supplementary file 1 — 10.1186/s12933-016-0414-5 Body weights of C57BL/6J mice fed a western diet (WD) high in fat and high fructose corn syrup compared to mice fed a control diet (CD). WD induced significant increases in body weight in WDC and WDL compared to their respective control groups. Administration of linagliptin (L) had no effect on body weight. [file 12933_2016_414_MOESM1_ESM.docx]

**Table S1.** Body weights of C57BL/6J mice fed a western diet (WD) high in fat and high fructose corn syrup compared to mice fed a control diet (CD). WD induced significant increases in body weight in WDC and WDL compared to their respective control groups. Administration of Linagliptin (L) had no effect on body weight.

| Body Wt | Main Effect | P value | CDC  (10) | CDL  (8) | WDC  (10) | WDL  (10) |
| --- | --- | --- | --- | --- | --- | --- |
| Pre-Treatment  (g) | Diet  Treatment  Interaction | 0.915  0.735  0.805 | 12.3  ±0.4 | 12.7  ±0.5 | 12.2  ±0.2 | 12.4  ±0.4 |
| Post-Treatment  (g) | Diet  Treatment  Interaction | 0.005  0.947  0.791 | 21.1  ±0.4 | 22.0  ±0.7 | 23.5*  ±0.7 | 23.4§  ±1.0 |
| Delta  (g) | Diet  Treatment  Interaction | 0.003  0.814  0.688 | 8.7  ±0.3 | 9.3  ±0.7 | 11.3*  ±0.6 | 10.9§  ±0.8 |
| % Increase | Diet  Treatment  Interaction | 0.006  0.700  0.677 | 71.3  ±7.7 | 74.1  ±6.9 | 96.8^a^  ±12.0 | 98.1§  ±7.1 |

Values are mean ± SE. Control Diet Control (CDC), Control Diet Linagliptin (CDL), Western Diet Control (WDC), and Western Diet Linagliptin (WDL). Post-hoc comparisons; *P<0.05 CDC vs WDC; § CDL vs WDL. ^a^ P=0.06.
